# A Method to Track 3D Knee Kinematics by Multi-Channel 3D-Tracked A-Mode Ultrasound

**DOI:** 10.3390/s24082439

**Published:** 2024-04-11

**Authors:** Kenan Niu, Victor Sluiter, Bangyu Lan, Jasper Homminga, André Sprengers, Nico Verdonschot

**Affiliations:** 1 Robotics and Mechatronics Group, Faculty of Electrical Engineering, Mathematics and Computer Science (EEMCS), University of Twente, P.O. Box 217, 7500 AE Enschede, The Netherlands; b.lan@student.utwente.nl; 2 Department of Biomechanical Engineering, University of Twente, 7521 HK Enschede, The Netherlandsj.j.homminga@utwente.nl (J.H.);; 3 Orthopaedic Research Lab, Radboud University Medical Center, P.O. Box 9101, 6500 HB Nijmegen, The Netherlands

**Keywords:** A-mode ultrasound, motion tracking, wearable ultrasound, gait analysis, tibiofemoral kinematics

## Abstract

This paper introduces a method for measuring 3D tibiofemoral kinematics using a multi-channel A-mode ultrasound system under dynamic conditions. The proposed system consists of a multi-channel A-mode ultrasound system integrated with a conventional motion capture system (i.e., optical tracking system). This approach allows for the non-invasive and non-radiative quantification of the tibiofemoral joint’s six degrees of freedom (DOF). We demonstrated the feasibility and accuracy of this method in the cadaveric experiment. The knee joint’s motions were mimicked by manually manipulating the leg through multiple motion cycles from flexion to extension. To measure it, six custom ultrasound holders, equipped with a total of 30 A-mode ultrasound transducers and 18 optical markers, were mounted on various anatomical regions of the lower extremity of the specimen. During experiments, 3D-tracked intra-cortical bone pins were inserted into the femur and tibia to measure the ground truth of tibiofemoral kinematics. The results were compared with the tibiofemoral kinematics derived from the proposed ultrasound system. The results showed an average rotational error of 1.51 ± 1.13° and a translational error of 3.14 ± 1.72 mm for the ultrasound-derived kinematics, compared to the ground truth. In conclusion, this multi-channel A-mode ultrasound system demonstrated a great potential of effectively measuring tibiofemoral kinematics during dynamic motions. Its improved accuracy, nature of non-invasiveness, and lack of radiation exposure make this method a promising alternative to incorporate into gait analysis and prosthetic kinematic measurements later.

## 1. Introduction

Measuring joint kinematics during daily activities is crucial for understanding both normal and pathological joint functions. Also, such measurements are valuable in assessing orthopedic surgery outcomes and their potential implications for the improvements of design joint implants. An accurate determination of skeletal kinematics necessitates the acquisition of reliable data that precisely represent bone motion [1]. At the knee joint, accurate tibiofemoral kinematics is essential for clinicians to understand, diagnose, and evaluate the behavior of the intact, diseased, or prosthetic knee.

To measure in vivo kinematics of the lower extremity, several researchers used intra-cortical bone pins with mounted markers [2]. The positions of these bone pins are tracked by a stereo photographic system (e.g., an optical tracking system). This approach offers a highly accurate representation of bone segment motions in the knee joint. However, its invasive nature impedes its clinical application. As an alternative, skin-mounted markers are widely used to measure the kinematics of the lower extremity for gait analysis, where the trajectories of skin markers represent the movements of the underlying bone segments [3,4,5]. This method, however, is constrained by its lower accuracy due to soft tissue artifacts (STAs) [6]. Fluoroscopic systems, achieving a high accuracy using radiographic imaging and sophisticated model-based methods [7,8,9], are another alternative. Nevertheless, the radiation exposure to the subject, the limited field of view, and the high cost limit their routine use in clinical settings.

The ultrasound (US) technique provides a non-invasive, radiation-free means to detect the tissue–bone boundary and estimate its depth through soft tissue during movement. It also enables the registration of ultrasound images to the segmented bone in computer-aided orthopedic surgery [10,11,12]. A method using a tri-plane B-mode (Brightness-mode) ultrasound has demonstrated the feasibility of estimating knee joint kinematics by combining multiple B-mode ultrasound transducers with an image registration algorithm [13]. However, A-mode transducers (i.e., single-element ultrasound transducers) are more cost-effective and smaller in size compared to B-mode transducers, and they are more accurate for biometric measurement, e.g., the depth of anatomical structures [14,15].

In this study, we introduced a real-time tibiofemoral kinematics measurement system to track knee joint movement, leveraging multiple A-mode ultrasound transducers integrated with a conventional motion capture system (i.e., optical tracking system). These transducers continuously measure the 3D spatial locations of the femur and tibia under dynamic conditions. We designed multiple customized ultrasound holders to attach to six anatomical regions of the lower extremity, facilitating the digitization of different bone reflection points during subject movement. These digitized points are used to reconstruct the 3D position of the femur with respect to the tibia. The objectives of this paper are twofold: firstly, to describe the approaches for measuring tibiofemoral kinematics, including data acquisition, signal processing, and tibiofemoral pose estimation; secondly, to demonstrate the feasibility of the proposed method and evaluate the accuracy of the measured kinematics. A cadaveric experiment was conducted using the proposed system to capture kinematics, and the results were compared to those obtained using intra-cortical bone pins tracked with a motion capture system.

## 2. System Design and Method

### 2.1. Data Acquisition

**Figure 1:**
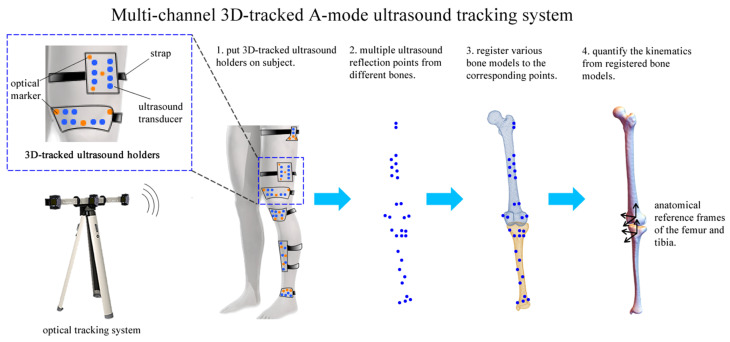
A system overview of the multi-channel A-mode ultrasound system to quantify tibiofemoral kinematics.

The multi-channel 3D-tracked A-mode ultrasound tracking system comprises an optical tracking system (VZ4000v tracking systems, PTI Phoenix Technologies Inc., Vancouver, BC, Canada) and six customized 3D-tracked ultrasound holders (Figure 1).

Each holder is equipped with three optical markers and a varied number of A-mode ultrasound transducers (Imasonic SAS, Voray/l’Ognon, France). The optical markers are responsible for providing spatial localization information, while the transducers detect the depth of the tissue–bone boundary. In total, this system utilizes 30 A-mode ultrasound transducers and 18 optical markers across the six holders. These transducers operate at a frequency of 7.5 MHz and are focused at a depth of 2.5 cm.

**Figure 2:**
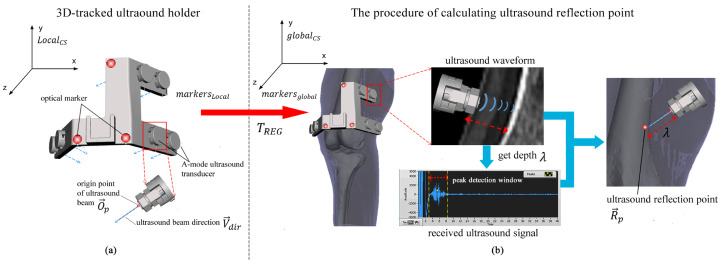
The methodology of calculating the ultrasound reflection point on the bone surface using a 3D-tracked ultrasound holder. The depth λ is determined from the received ultrasound signal by establishing a peak detection window. The ultrasound reflection point (${\vec{R}}_{p}$) is identified by determining the origin point of the ultrasound beam (${\vec{O}}_{p}$), the unit vector indicating the direction of the ultrasound beam (${\vec{V}}_{dir}$), and the transformation ($T_{REG}$). This transformation aligns three optical markers from the local holder coordinate system with the global coordinate system.

The design of the 3D-tracked ultrasound holders was carried out in SolidWorks 2016 (Waltham, MA, USA) and was tailored to the anatomical dimensions of a subject to ensure optimal skin contact for each A-mode ultrasound transducer. The holders were manufactured using a 3D printing device (EOS Formiga P110, EOS GmbH, Krailling, Germany) and Polyamide powder material. The high precision of the 3D printing process, together with the rigid design of the holders, ensured that the spatial relationship between the optical markers and the A-mode ultrasound transducers remained consistent throughout all measurements. During the design phase, for each A-mode ultrasound transducer, the origin point ($\vec{O}$) and the unit pointing direction (${\vec{V}}_{dir}$) of the ultrasound beam were calculated in the local holder coordinate system ($Local_{CS}$). Concurrently, the 3D locations of the three optical markers ($markers_{Local}$) for each ultrasound holder were digitized in the $Local_{CS}$. During measurement, the 3D locations of these optical markers for each holder were recorded in the global coordinate system ($global_{CS}$), denoted as $markers_{global}$. The rigid-body transformation from $markers_{Local}$ to $markers_{global}$ of each ultrasound holder was found using the point-to-point registration method described in [16], termed $T_{REG}$. Thus, an ultrasound refection point (${\vec{R}}_{p}$) could be digitized when the depths (λ) were obtained from the received ultrasound signals (Figure 2).

(1) $${\vec{R}}_{p}=T_{REG}\left({\vec{O}}_{p}+\lambda {\vec{V}}_{dir}\right)$$

### 2.2. Working Principle

The working principle of the multi-channel 3D-tracked A-mode ultrasound tracking system is depicted in Figure 2. This system employs six customized 3D-tracked ultrasound holders, each attached to specific anatomical areas: the greater trochanter, the middle part of the femur, the distal femur, the proximal tibia, the middle part of the tibia, and the ankle. These holders are simultaneously tracked by an optical tracking system. As the subject moves, ultrasound echos are received from the femur and tibia to digitize multiple ultrasound reflection points. Subsequently, segmented bone models of the tibia and femur, derived from CT scans, are instantaneously registered to these ultrasound reflection points using registration algorithms [12,15,17]. This process enables the determination of knee joint kinematics by analyzing the positions of the registered bone models. Consequently, the system allows for the continuous monitoring of the relative positions of the registered femur and tibia. This facilitates the quantification of rotations and translations between these two segments in real time during dynamic motion, as illustrated in Figure 2.

### 2.3. Ultrasound Signal Processing

In the process of ultrasound signal processing, the received signal of each A-mode ultrasound transducer was filtered using a second-order Butterworth filter with a cut-off frequency of 2 MHz. Prior to dynamic measurements, a specific peak detection window was manually established for each transducer within the received ultrasound waveform. The calibration of this window was based on the ultrasound signals captured while flexing the subject’s knee joint in advance. This preparatory step was crucial, as it facilitated faster and more reliable peak detection within the most probable range of the tissue–bone boundary for different transducers. During the dynamic measurements, a peak detection method was applied. The peak that has a greater amplitude than the setting threshold and the biggest slope was determined as the target peak representing the tissue–bone boundary.

### 2.4. Tibiofemoral Pose Estimation

After attaching all ultrasound holders on the lower extremity, 30 ultrasound reflection points could be digitized concurrently as the subject moved. Among all points,15 points were selected for the femur and tibia, including key anatomical landmarks, such as the lateral and medial epicondyles, great trochanter, and ankle joint [18,19]. As the A-mode ultrasound has limitations in the depth of penetration and the field of view, to ensure effective ultrasound wave penetration and the high-quality raw signal received, the selection of the remaining anatomical sites was guided by the ease of obtaining valid bone reflections at various flexion angles. These captured points were fed to a registration algorithm that combined an iterative closest point algorithm [20] and perturbation method [21], allowing for the registration of bony segments to the corresponding ultrasound reflection points for each time frame. The registration was performed by providing an initial guess of the pose of an object and following an optimization that minimizes the cost function of point-to-object matching. The duration of the preparation and analysis time was about 1 h, including the attachment of ultrasound holders to the subject, the calibration procedure, and the measurement.

In our case, we used key anatomical landmarks to establish this initial guess (pre-registration), such as the lateral/medial femoral epicondyles, tibia epicondyles, the greater trochanter, and the ankle. A subsequent optimization was undertaken using a modified weighted iterative closest point algorithm [22]. This algorithm’s weighting factors were associated with the intensity (i.e., ‘reliability’) of the detected peak at the tissue–bone boundary. The output of the registration algorithm was a transformation matrix that precisely aligned the segmented bone model with the acquired ultrasound refection points. After applying the transformation matrix to the segmented bone model, we could estimate the 3D position and orientation of the bone model (i.e., the position of the registered bone model). When the anatomical coordinate systems of the femur and tibia were determined from the segmented bone models [23], the tibiofemoral kinematics could be calculated from the estimated positions of the registered femur and tibia per time frame using the method from [24].

### 2.5. Cadaver Experiment

To demonstrate the feasibility and accuracy of our system, we conducted a comprehensive cadaver experiment. For this purpose, a full-body male cadaver specimen (79 kg, 179 cm) was obtained from and approved by the Anatomy Department of the Radboud Medical Center (RUMC), Nijmegen, The Netherlands. This specimen had no known history of illness, injury, or treatment affecting the knee or hip joints.

**Figure 3:**
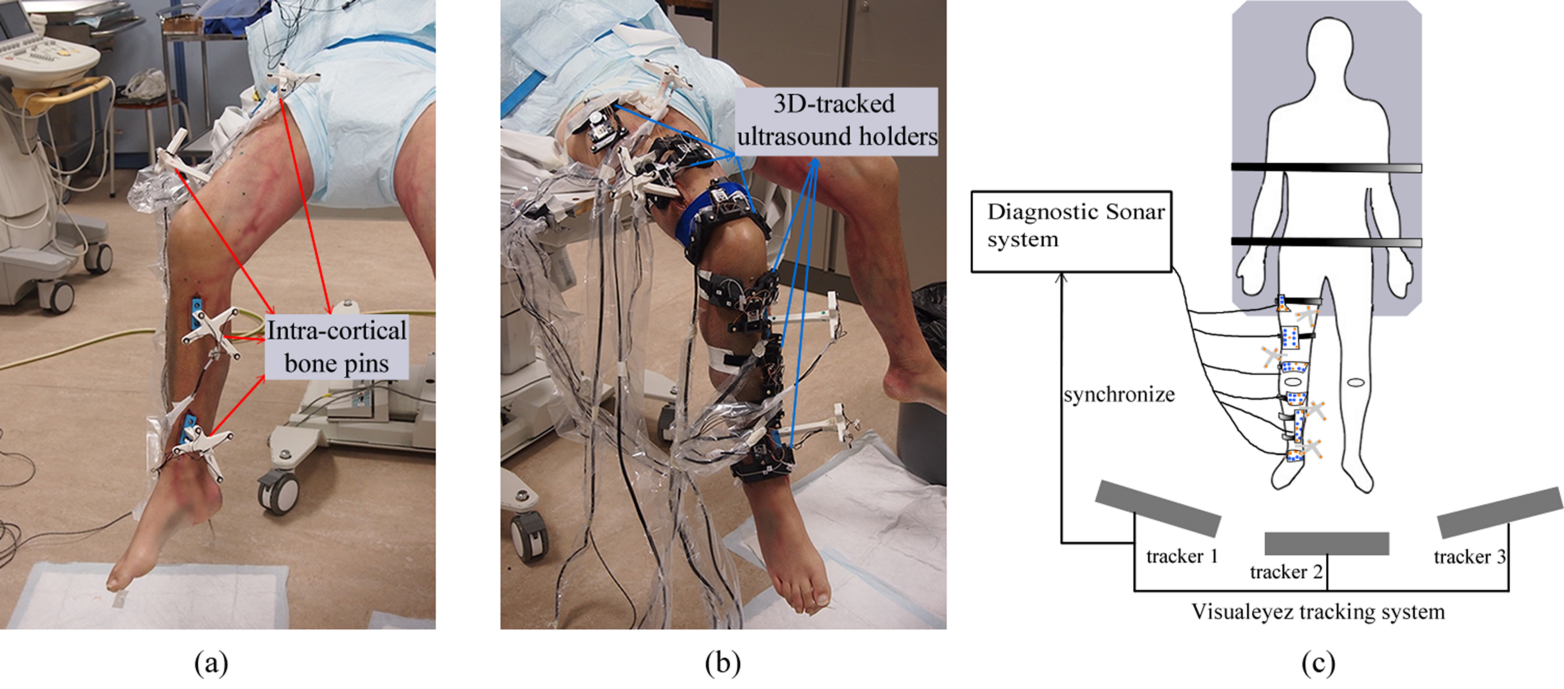
(**a**) Four intra-cortical bone pins was inserted into the lower extremity to produce gold-standard kinematics, two in the femur and two on the tibia. (**b**) Six 3D-tracked ultrasound holders attached to the lower extremity produced the ultrasound-derived kinematics. (**c**) A schematic of a cadaver experiment showing the multi-channel A-mode ultrasound tracking system comprised of Visualeyez tracking system and Diagnostic Sonar system.

Prior to the experiment, dimension measurements were taken in various anatomical regions for designing subject-specific ultrasound holders, including the circumferences of the mid-thigh and shank, the distal femur, the proximal tibia, and the ankle. In addition, two intra-cortical bone pins, each equipped with four optical markers, were inserted into the femur and tibia separately (four intra-cortical bone pins and 16 optical markers in total) to track the actual motion of the leg and establish gold-standard kinematics (Figure 3a). Following the insertion of the bone pins, a CT scan was performed at the RUMC’s Department of Radiology using a TOSHIBA Aquilion ONE scanner (TOSHIBA, Tustin, CA, USA), with a voxel size of 0.755 mm × 0.755 mm × 0.500 mm. The femur and tibia bone models were manually segmented from these CT images to generate a surface mesh in STL format using Mimics 17.0 software. The 3D locations of the optical markers mounted on the bone pins were also digitized in the CT image coordinate system.

**Figure 4:**
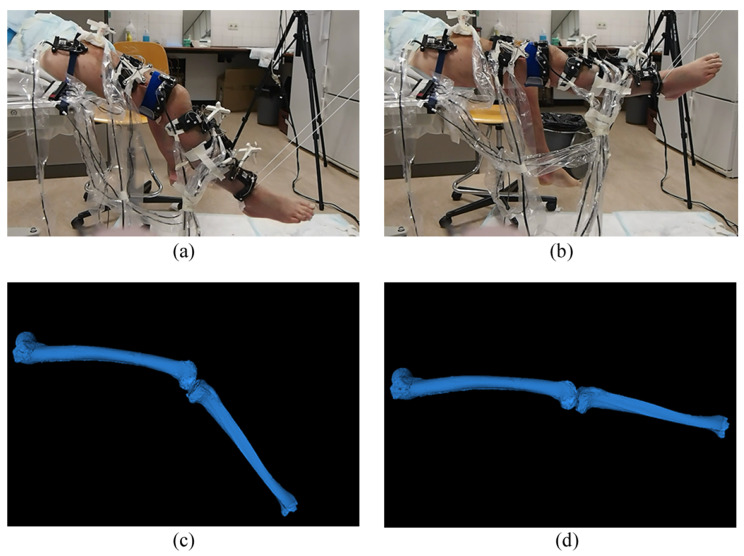
(**a**) The knee joint of the specimen was kept at a certain flexion angle. (**b**) The knee joint of the specimen was moved to full extension. (**c**) The estimated 3D positions of the registered femur and tibia at a flexion angle corresponding to (**a**). (**d**) The estimated 3D positions of the registered femur and tibia at the same extension pose as (**b**).

During the experiment, the upper body of the cadaver was secured to a surgical table using nylon straps. The right leg’s flexion angle was manually cycled between flexion and extension to simulate the swinging phase of walking (Figure 4a,b). This cycle, consisting of seven total motions, started with flexion, extended to the endpoint, and then flexed back to the starting position. Six custom A-mode ultrasound holders were mounted on the lower extremity (Figure 3b). All optical markers (16 from the bone pins, 18 from the ultrasound holders) were concurrently tracked by the Visualeyez optical tracking system (VZ4000v, PTI Phoenix Technologies Inc., Vancouver, BC, Canada) using three calibrated trackers operating at 60 Hz (Figure 3c). The ultrasound signals were captured and synchronized with the Visualeyez tracking system using the Diagnostic Sonar FI Toolbox (Livingston, Scotland), powered by a 2.3 GHZ CPU (Intel Core i7-3610QE) and 8 GB of RAM. The acquisition sample rate was set as 25 Hz. Custom software developed in LabVIEW 2015 (National Instruments, Austin, TX, USA) provided a graphical user interface for data storage and post-processing. To assess the accuracy of our proposed system, we compared the gold-standard tibiofemoral kinematics derived from inserted bone pins with the ultrasound-derived kinematics. The comparison involved calculating the mean, standard deviation (SD), and root-mean-square (RMS) errors of the absolute differences between these two sets of kinematics.

To visually represent the differences between the two sets of kinematics, the 6-degrees-of-freedom (DOF) kinematics were plotted as a function of an increasing sample number. Given that the cadaveric leg was manipulated in a periodic manner, we also segmented the full kinematic data into seven distinct cyclic motions, each beginning with flexion, moving to extension, and returning to flexion to complete the cycle. The lengths of segmented cyclic motions were normalized in order to illustrate 6 DOF kinematics with the corresponding percentage of cyclic motion.

## 3. Results

**Figure 5:**
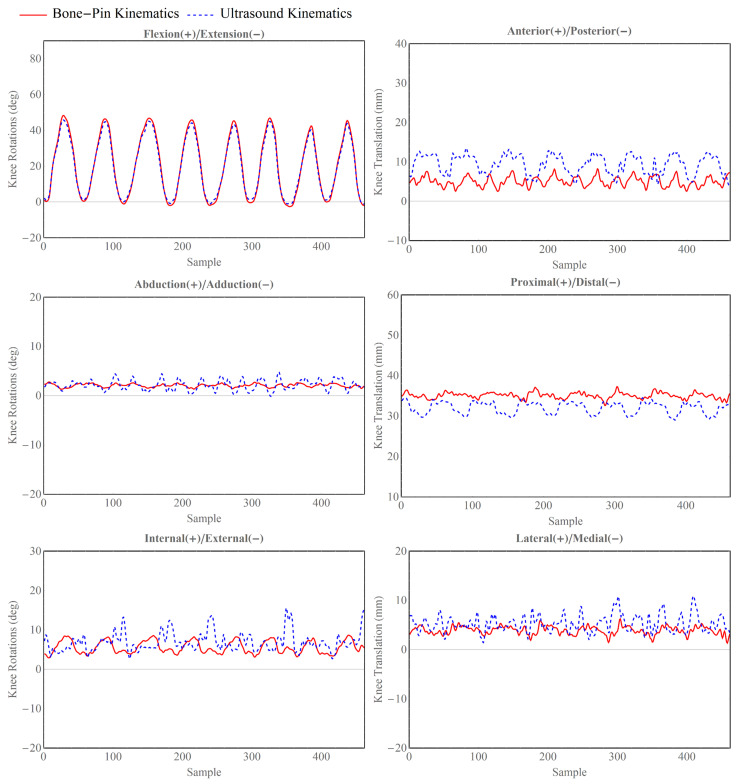
Comparisons of the ultrasound-derived tibiofemoral kinematics (blue dashed lines) and gold-standard kinematics derived from bone pins (red, solid line) on knee joint flexion(+)/extension(−), adduction(+)/abduction(−), and external(+)/internal(−) rotations and anterior(+)/posterior(−), proximal(+)/distal(−), and lateral(+)/medial(−) translations.

**Figure 6:**
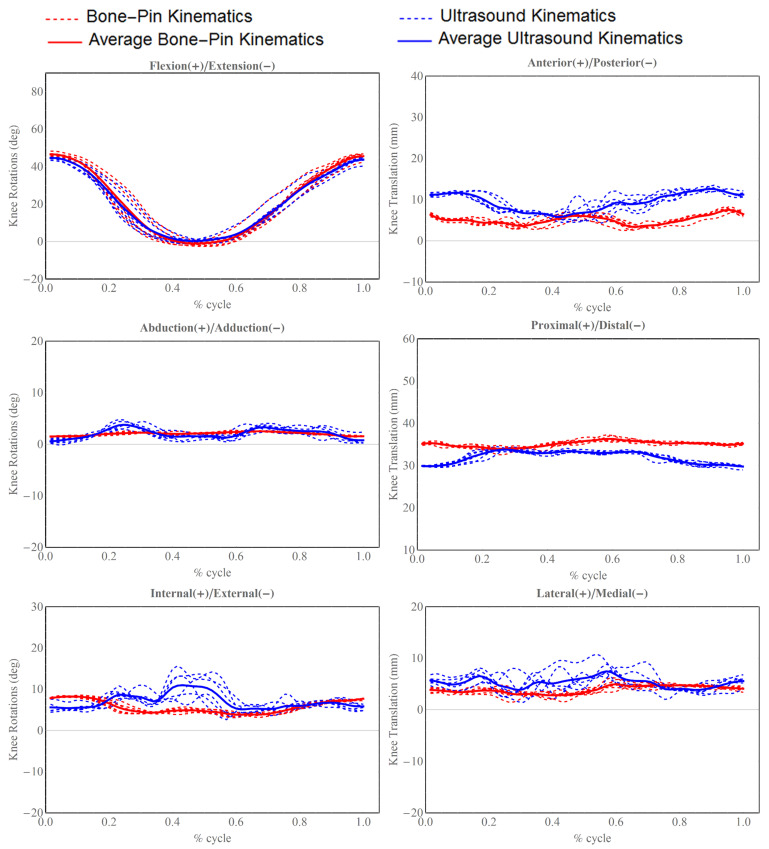
Six DOF tibiofemoral kinematics derived from ultrasound and bone pins are displayed in cyclical fashion. All cyclical trials of ultrasound-derived kinematics are plotted in blue dash lines, and the solid thick blue line represents average motion cycle estimated from ultrasound system; all bone pins trials are plotted in dashed red lines, and solid thick red line represents the average motion cycle measured from bone pins.

The experiment results are illustrated in Figure 5 and Figure 6. The patterns observed in flexion(+)/extension(−) rotation, adduction(+)/abduction(−) rotation, and lateral(+)/medial(−) were found to closely align with the trends of the gold-standard kinematic components. For the anterior(+)/posterior(−) and proximal(+)/distal(−) translations, there were systematic errors between the gold-standard and ultrasound-derived kinematics.

In terms of accuracy, the root-mean-square (RMS) errors for joint rotations ranged from 0.88° to 3.28°. For joint translations shown in Table 1, the RMS errors spanned from 2.29 mm to 5.04 mm. The most significant rotational error was associated with the external–internal rotation, whereas the anterior–posterior translation exhibited the largest translational error. On average, the rotational errors in the ultrasound-derived kinematics were 1.51 ± 1.13° (mean ± standard deviation [SD]), and the translational errors were 3.14 ± 1.72 mm (mean ± SD) when compared to the gold-standard measurements.

## 4. Discussion

This study introduces an alternative method of tibiofemoral kinematics measurement using a multi-channel 3D-tracked A-mode ultrasound approach. The feasibility and accuracy of this method were validated through a cadaver experiment. Our findings demonstrate the system’s capability to measure 3D knee joint motion dynamically during simulated flexion–extension movements. This new approach integrated two existing techniques: 3D motion capture and ultrasound technology. This combination yields a non-invasive, radiation-free method that still maintains a relatively high accuracy. It is non-invasive and without radiation and at the same time provides a relatively high accuracy. Our initial goal was to achieve kinematic measurement accuracy within 1 mm and 1° for translational and rotational errors, respectively, aspiring to parallel the performance of state-of-the-art biplane fluoroscopic systems [9,25,26]. This system holds potential as an alternative for measuring prosthetic kinematics in total knee arthroplasty (TKA) patients and for gait analysis.

The smallest RMS error recorded was 0.88° for abduction(+)/adduction(−), achieving sub-degree accuracy comparable to fluoroscopic systems, which report a 0.77° RMS error [27]. The maximum RMS errors observed were 3.28° and 5.04 mm for external(+)/internal(−) rotation and anterior(+)/posterior(−) translation, respectively. The pronounced translational error could be attributed to a systematic deviation during the registration process, indicating a need for further research to refine this aspect and achieve the required accuracy for prosthetic kinematic measurements.

Compared to skin-mounted markers, our multi-channel A-mode ultrasound system potentially offers more accurate tibiofemoral kinematics, as it estimates the bone position and orientation directly from bony points rather than from skin-mounted markers. This approach could effectively mitigate the effects of soft tissue artifacts (STAs). In systems using skin-mounted markers, STAs are typically present and challenging to eliminate due to the inherent mismatch between skin and bone movement [28,29,30]. For instance, the reported accuracy of such systems for walking motion tasks shows average errors up to 4.40° and 13.0 mm for rotations and translations, respectively [31]. In contrast, the largest mean errors in our system were 2.46° and 4.55 mm, demonstrating a lower error margin than that reported for skin markers. However, a more detailed comparison is required to fully evaluate our system’s performance against skin-mounted marker systems. This part of this work has been reported in [4]. While flexion/extension and abduction/adduction rotations were relatively comparable in accuracy to fluoroscopic systems, other motions exhibited larger errors. Therefore, our future research aims to enhance the current system’s accuracy to match our desired goals and to achieve errors comparable to those of fluoroscopic systems.

There are several factors contributing to the error observed in our ultrasound system. Firstly, the loss of sight between the ultrasound probe and bone during movement is a critical issue, as the bone may move out of the ultrasound probe’s field. Secondly, the incorrect detection of the reflection peak is a significant error source, directly related to the varying distance between the bone and ultrasound probe and the sensitivity to soft tissue variability. Thirdly, inherent errors in the registration algorithm contribute to inaccuracies. Lastly, the lack of geometric constraints in the distal–proximal direction also leads to measurement errors.

The first and second factors are interrelated and strongly influenced by the incident angle of the A-mode ultrasound transducer, affecting both the optimal field of view and the quality of the ultrasound echo signal [32,33]. Maintaining the transducer perpendicular to the bone surface facilitates an easier detection of the bone peak in the ultrasound echo signal [18,34]. However, consistently achieving this perpendicular angle during movement is challenging. Additionally, the positioning of the specimen on the surgical table caused compression of soft tissues at certain points, abnormally increasing the depth of the tissue–bone boundary, a situation that differs from normal soft tissue deformation during standing or walking. In addition, the bone to transducer distance was determined from the ultrasound echo signal. Sometimes, an incorrect peak is selected, leading to inaccurate bone reflection points in the automatic registration algorithm. Future efforts will focus on ensuring the continuous visibility of the bone signal in A-mode ultrasound echoes during movement and developing more precise and reliable peak detection methods.

The third and fourth factors, related to the registration algorithm error and the lack of geometric constraints, are closely connected. In our initial prototype, we utilized 30 A-mode ultrasound transducers. This number was a limitation, particularly as the mid-shaft areas of the femur and tibia, being largely symmetric along the longitudinal axis, tended to exhibit greater errors in this direction than in others. To address this, future developments will include increasing the number of transducers and repositioning them on newly designed custom holders to better cover various anatomical regions. A more advanced registration algorithm will be adopted to improve registration accuracy in our scenario.

This study presents the feasibility and utility of using ultrasound in kinematic analysis. Similarly, surface electromyography (sEMG) has long been used for analyzing muscle activity. It has been leveraged in recent years for kinematics estimation, especially when integrated with other sources of information [35,36]. The integration of ultrasound and sEMG technologies enables a more comprehensive kinematic analysis, which is promising for the development of intelligent prosthetic controllers. Moreover, using ultrasound, one study [37] showcased the capability to predict walking kinematics of transfemoral amputee prostheses by artificial neural networks. This study paves the way to better, advanced ultrasound-based predictions in kinematic studies.

In addition, although the tracking and registration process can be in real time, the preparation time required is 1 h, which limits the end-to-end real-time application. In addition, proper calibration is essential for ensuring that the A-mode US system accurately reflects the anatomical positions and orientations. The wearability of the system requires that the system does not inhibit natural movements, especially during dynamic activities.

Therefore, designing a calibration procedure and a wearable A-mode US system that is both accurate and user-friendly should be further discussed and investigated. For better validation of the system during in vivo situations, a wider range of movements and conditions should be tested in future study, including various patient populations and orthopedic conditions for better assessing the diagnostic utility of the technique. This helps to overcome any potential technical challenges and improve the system’s accuracy and usability.

This study possesses several notable strengths. First and foremost, the use of intra-cortical bone pins as a gold standard for kinematics, measured concurrently with all 3D-tracked ultrasound holders, effectively eliminates synchronization issues often encountered with different tracking modalities. Secondly, the customized 3D-tracked ultrasound holders were manufactured using an accurate 3D printing process, which simplified the procedure of calibrating all ultrasound holders. Furthermore, the operational principle of 3D-tracked ultrasound holders is well suited for clinical applications, offering simplicity and usability. While the current system may not match the accuracy of most fluoroscopic systems in reconstructing kinematics, it offers a non-invasive, radiation-free method for reconstructing tibiofemoral kinematics. With significant potential for enhancements in the overall system design, registration algorithms, and peak detection methods, we expect to develop a system whose accuracy in measuring tibiofemoral kinematics rivals that of fluoroscopic systems.

## 5. Conclusions

This study presents a multi-channel A-mode ultrasound system and validates its feasibility for tracking tibiofemoral kinematics by a cadaver experiment. Although the accuracy comparison showed that the reconstructed tibiofemoral kinematics were less precise than those reported in the fluoroscopic systems, this system shows significant promise in overcoming the limitations posed by soft tissue artifacts associated with skin-mounted marker systems. Also, it offers a non-invasive and radiation-free alternative for measuring tibiofemoral kinematics. Thus, this development holds considerable promise for enhancing clinical gait analysis and improving the accuracy of prosthetic kinematic assessments in the future.

## Figures and Tables

**Table 1:** Mean, standard deviation (SD), and root-mean-square (RMS) errors of absolute differences between the ultrasound-derived tibiofemoral kinematics and bone pin kinematics (gold standard): flexion–extension (Flex/Ext), adduction–abduction (Add/Abd), and external–internal (Ext/Int) rotations and anterior–posterior (Ant/Post), proximal–distal (Prox/Dist), and lateral–medial (Lat/Med) translations.

Joint Rotational Errors (°)	Joint Translational Errors (mm)				
	Flex/Ext	Add/Abd	Ext/Int	Ant/Post	Prox/Dist
Mean	1.32	0.71	2.49	4.55	3.08
SD	0.73	0.52	2.14	2.17	1.57
RMS	1.51	0.88	3.28	5.04	3.45

## Data Availability

Data are contained within the article.

## Abbreviations

The following abbreviations are used in this manuscript:

US	Ultrasound
DOF	Degrees of freedom
STA	Soft tissue artifact
SD	Standard deviation
RMS	Root mean square
Flex	Flexion rotation
Ext	Extension rotation
Add	Adduction rotation
Abd	Abduction rotation
Ext	External rotation
Int	Internal rotation
Ant	Anterior translation
Post	Posterior translation
Prox	Proximal translation
Dist	Distal translation
Lat	Lateral translation
Med	Medial translation
